# m^6^A modification in immune-cell inflammatory fate: from RNA fate regulation to therapeutic translation in inflammatory diseases

**DOI:** 10.3389/fimmu.2026.1918662

**Published:** 2026-08-27

**Authors:** Yizhang Zhu, Shiye Yang, Lei Qin, Dongyang Ding, Xiaojie Gan

**Affiliations:** 1 Department of General Surgery, The First Affiliated Hospital of Soochow University, Suzhou, China; 2 Department of Comprehensive Surgery, Affiliated Nantong Clinical College of Nantong University, Nantong First People’s Hospital, Nantong, Jiangsu, China; 3 The Third Department of Hepatic Surgery, Eastern Hepatobiliary Surgery Hospital, Naval Medical University, Shanghai, China

**Keywords:** immune cells, immunometabolism, inflammation resolution, inflammatory diseases, m^6^A, RNA fate

## Abstract

Inflammatory disease is sustained not only by immune activation but also by the failure of activated cells to terminate effector programs and return tissues to homeostasis. This review evaluates N^6^-methyladenosine (m^6^A) as a post-transcriptional regulator of that persistence. Rather than cataloguing diseases or classifying individual m^6^A regulators as pro- or anti-inflammatory, we organize the evidence around a causal chain linking a regulator and target RNA to a site- or reader-dependent change in RNA fate, an immune-cell phenotype, and a disease outcome. Mechanistically developed evidence is concentrated in macrophages and T cells, including STAT1 stability and decay, SOCS turnover, SLC37A2 translation, and lineage-specific T-cell programs. Mechanisms in other innate and adaptive immune populations are emerging, but cell-subset resolution and disease-stage validation remain uneven. Across inflammatory signaling, immunometabolic, and cell-death pathways, the same m^6^A regulator can produce opposing outcomes through different target transcripts or readers. We therefore distinguish mechanistically resolved, functional/intermediate, and associative evidence while highlighting the limitations of global m^6^A assays, bulk-tissue profiling, and pathway-level inference. The translational value of m^6^A is likely to depend on target-RNA selection, cell-specific delivery, disease-stage timing, and preservation of protective immunity and tissue repair. An evidence-aware, transcript-centered framework may help move m^6^A research from descriptive association toward causal immune-state biology and clinically testable interventions.

## 1 Introduction

Inflammation is indispensable for host defense and tissue repair, but it becomes pathogenic when immune activation does not contract after the initiating threat has been controlled. This failure of resolution is shared by otherwise distinct conditions, including rheumatoid arthritis (RA), systemic lupus erythematosus (SLE), inflammatory bowel disease (IBD), atherosclerosis, obesity-associated inflammation, metabolic dysfunction-associated steatotic liver disease (MASLD), sepsis, and chronic barrier-tissue disorders (1, 2). Across these settings, the key biological problem is not simply that immune cells are activated; it is that selected cell states remain abnormally stable, continue to recruit or instruct other cells, and resist the negative-feedback programs that normally restore homeostasis.

Most mechanistic models of inflammatory persistence emphasize cytokines, transcription factors, and canonical pathways such as NF-κB, JAK/STAT, the NLRP3 inflammasome, and HIF-1α. These pathways are essential, but transcription alone does not explain how immune cells alter protein output within minutes, maintain a response after the initiating signal has weakened, or rapidly reverse that response during resolution. Such behavior depends on post-transcriptional control of RNA stability, translation, splicing, nuclear export, localization, and degradation (3–5). RNA fate is therefore not a secondary layer of regulation; it is a central determinant of the amplitude, duration, and reversibility of inflammatory programs.

N^6^-methyladenosine (m^6^A) is one of the most prevalent internal modifications of eukaryotic messenger RNA. It is installed by a writer complex containing METTL3, METTL14, WTAP, and associated cofactors; removed by the demethylases FTO and ALKBH5; and interpreted by readers such as YTHDF, YTHDC, and IGF2BP proteins (6, 7, 20, 21). The functional consequence of an m^6^A mark is not fixed. Depending on the modified transcript, the position of the mark, the available reader, and the cellular state, m^6^A can accelerate RNA decay, increase translation, stabilize an RNA, alter splicing, or change subcellular localization. This target- and reader-dependence is particularly important in inflammation, because the same regulator can support a protective response through one transcript while amplifying pathology through another.

Previous reviews have catalogued m^6^A regulators across inflammatory diseases or immune-cell types (8–14, 23, 24). The present review takes a different, evidence-aware approach. The principal contribution of this review is not another catalogue of m^6^A regulators, but an evidence-oriented framework for judging whether reported associations support causal, cell-state-specific mechanisms. We organize the literature around a causal chain—m^6^A regulator, direct target RNA, site or transcript region, reader-dependent RNA-fate change, immune-cell phenotype, and disease-level consequence—and explicitly distinguish mechanistically resolved findings from functional but incomplete evidence and from hypotheses that remain largely associative. We focus on how m^6^A may stabilize or destabilize inflammatory cell states, compare areas with strong evidence (particularly macrophages and T cells) with less mature fields (neutrophils, dendritic cells, NK cells, ILCs, and B cells), and identify the experimental standards required for therapeutic translation.

## 2 Conceptual and evidence framework: linking m^6^A-dependent RNA fate to inflammatory cell-state persistence

Inflammatory stimuli—including pathogen-associated molecular patterns (PAMPs), damage-associated molecular patterns (DAMPs), cytokines, lipid metabolites, hypoxia, oxidative stress, and mechanical injury—first alter receptor signaling and transcription. m^6^A-dependent RNA control then acts on the transcripts generated or mobilized by those signals. By changing their stability, translation, localization, or decay, m^6^A can modify activation thresholds, metabolic adaptation, lineage stability, cell survival, and the capacity to return to quiescence. In this model, m^6^A does not independently “cause inflammation.” Rather, it determines how efficiently a cell converts an inflammatory input into protein output and how long that output persists after the input changes (15–24).

The writer–eraser–reader terminology is useful but can encourage an overly regulator-centric interpretation. Measuring increased METTL3 or decreased FTO does not establish which RNA has been modified, whether the modification is functional, or which reader executes the effect. A causal m^6^A mechanism requires several connected observations: a defined transcript and, ideally, a mapped site or region; a change in methylation after perturbing the regulator; altered RNA stability, translation, splicing, or localization; reader dependence when relevant; and a functional phenotype that can be rescued by manipulating the target RNA or the modified site. Without this chain, altered regulator expression may be a downstream marker of inflammation rather than a driver of immune-cell fate (20, 21, 25).

This distinction also explains apparently conflicting findings. METTL3, for example, can promote macrophage inflammation by stabilizing or enhancing the translation of pro-inflammatory transcripts, yet restrain inflammation when its principal target supports negative feedback or metabolic homeostasis (26–30, 38–43). Similarly, YTHDF1 can enhance NLRP3 translation in one model but suppress NLRP3-dependent pyroptosis indirectly by increasing WWP1 in another (117, 118). These observations do not imply that the m^6^A system is internally inconsistent. They indicate that biological direction is encoded at the level of the target transcript, reader, cell subset, stimulus, and disease stage—not by the name of the m^6^A regulator alone.

**Figure 1:**
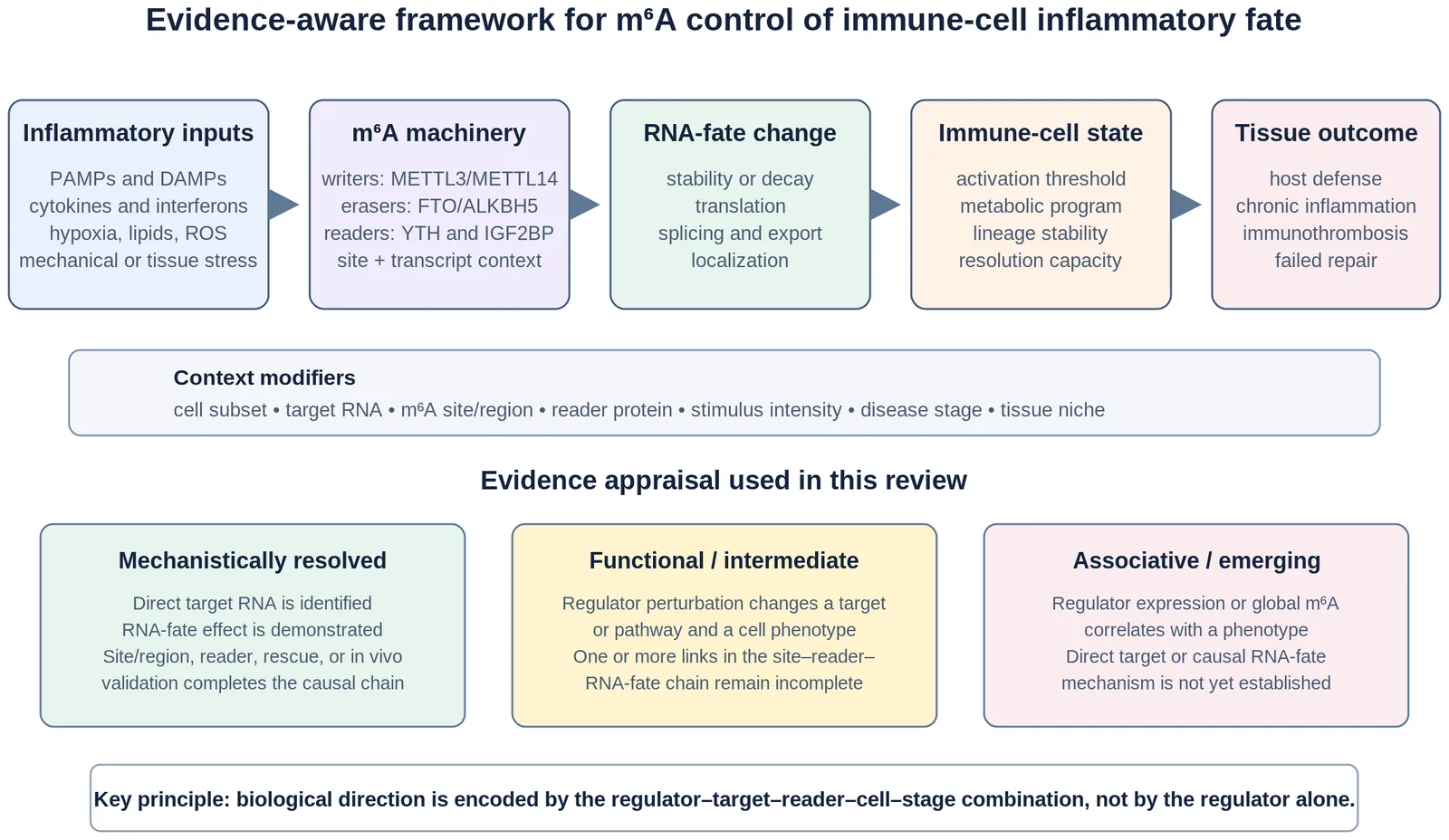
Evidence-aware framework for m^6^A control of immune-cell inflammatory fate. Inflammatory inputs are translated through writer, eraser, and reader proteins into transcript-specific changes in RNA stability, decay, translation, splicing, export, or localization. These RNA-fate changes can alter activation thresholds, metabolic programs, lineage stability, and resolution capacity, thereby influencing host defense, chronic inflammation, immunothrombosis, and failed repair. The biological direction of an m^6^A effect depends on cell subset, target RNA, modified site or transcript region, reader availability, stimulus intensity, disease stage, and tissue niche. Evidence is categorized as mechanistically resolved, functional/intermediate, or associative/emerging.

To make this heterogeneity explicit, we use three evidence categories throughout the review. “Mechanistically resolved” evidence identifies a direct target RNA and a defined RNA-fate mechanism, supported by site- or region-level mapping and, where applicable, reader-dependence, rescue experiments, or cell-specific *in vivo* validation. “Functional/intermediate” evidence links regulator perturbation to a target pathway and immune phenotype but leaves part of the site–reader–RNA-fate chain unresolved. “Associative/emerging” evidence consists mainly of expression correlations, global m^6^A changes, or plausible pathway extrapolation without direct causal validation. These categories are not formal quality scores; they are a transparent way to prevent well-established mechanisms and early hypotheses from being presented with equal certainty. This conceptual framework is summarized in **Figure 1**, which links inflammatory stimuli, m6A machinery, RNA fate regulation, immune-cell state transitions, and tissue outcomes, while explicitly grading evidence strength and emphasizing that biological direction is encoded by the regulator–target–reader–cell–stage combination rather than by any single regulator alone.

## 3 Innate immune cells: from danger sensing to persistent inflammatory programs

### 3.1 Macrophages: a comparatively well-developed evidence base, but not a single pro-inflammatory program

Macrophages are among the most extensively studied immune-cell populations in the inflammatory m^6^A literature. Their behavior is often summarized as M1 versus M2 polarization, but diseased tissues contain overlapping states that combine inflammatory cytokine production, lipid uptake, antigen presentation, angiogenesis, extracellular-matrix remodeling, efferocytosis, and repair (34–37). Accordingly, the relevant question is not whether m^6^A globally favors an M1 or M2 identity. It is which transcript-specific m^6^A events stabilize a particular macrophage program under a defined environmental pressure.

The METTL3–STAT1 axis provides one of the better-characterized mechanistic examples. METTL3-dependent methylation of STAT1 mRNA in the coding sequence and 3′ untranslated region has been reported to increase STAT1 RNA stability and activity, promote glycolytic enzyme expression, and support an inflammatory macrophage phenotype after IFN-γ or oxidized LDL stimulation (28, 104, 107). The opposite direction can be produced at the same transcript: RBM4 facilitates YTHDF2 recognition of m^6^A sites in the STAT1 3′ untranslated region, accelerating STAT1 decay and reducing STAT1-dependent glycolysis and M1-like polarization (105). More recently, a METTL3/RBM15–Kdm6b axis was linked to STAT1-associated macrophage activation and atherosclerosis (106). Together, these studies illustrate that the outcome depends on whether an m^6^A-marked transcript is stabilized, translated, or routed to decay.

Other direct axes connect m^6^A to macrophage metabolism and inflammatory feedback. IGF2BP2 stabilizes TSC1 and PPARγ transcripts and thereby influences macrophage activation and lipid handling (38). METTL3/YTHDF1-dependent translation of SLC37A2 has been reported to restrain LPS-driven glycolysis and intestinal inflammation, providing a counterexample to the common assumption that increased writer activity is necessarily pro-inflammatory (26). Conversely, METTL3-dependent regulation of HDGF and TIM1, and METTL14-associated regulation of MyD88/NF-κB signaling, have been linked to inflammatory activation in atherosclerotic or metabolic models (29, 39–41). DDX5-mediated modulation of m^6^A on TLR2/4 transcripts and METTL3-dependent effects on IRAKM further show that m^6^A can alter either receptor signaling or its termination (42, 43).

The macrophage literature is therefore mechanistically informative but still uneven. Several studies rely on immortalized lines, supraphysiological LPS exposure, or global overexpression and knockdown. “M1/M2” marker panels are often used as the primary endpoint even when the disease-relevant state is a lipid-associated, scar-associated, or tissue-resident macrophage. Stronger studies should combine site-resolved m^6^A mapping with primary cells, target-RNA rescue, myeloid-specific genetic models, and single-cell or spatial analysis in patient tissue. These approaches are needed to determine whether an m^6^A event initiates a macrophage state, merely maintains it, or reflects an already established inflammatory environment.

### 3.2 Neutrophils and NETosis: emerging direct mechanisms, but substantial disease-stage uncertainty

The evidence for m^6^A regulation in neutrophils is newer and less extensive than that in macrophages, but it is no longer purely speculative. ALKBH5 has been shown to support efficient antibacterial neutrophil migration, and subsequent work linked ALKBH5 to emergency granulopoiesis and mobilization through increased G-CSF receptor expression (54, 55). These studies support a functional role in neutrophil production and trafficking. They do not, however, establish that enhancing ALKBH5 would be beneficial in all inflammatory diseases, because the same increase in neutrophil recruitment that improves bacterial defense may aggravate sterile injury or immunothrombosis. Given their recent emergence and limited disease contexts, these mechanisms require independent replication in primary human neutrophils and additional disease models. Direct evidence connecting m^6^A to NET formation is beginning to emerge. METTL3 has been reported to promote NETosis through SYK/ERK/MEK signaling in acute lung injury, and histone lactylation-induced WTAP expression has been linked to TLR2-dependent inflammatory responses in neutrophils (56, 57).

An ALKBH5–m^6^A–LINC00968 axis has also been proposed to restrain oxidative-stress-driven NETosis in RA, whereas reduced YTHDF2 expression in SLE is associated with inflammatory and neutrophil cytokine signatures (58, 59). These findings are important, but they vary in mechanistic depth: some identify a target axis and functional NET phenotype, whereas others remain associative or are embedded in multi-component pharmacological interventions.

NETosis itself is context-dependent. During early infection, NETs can restrict pathogen dissemination; during prolonged sterile inflammation, autoimmunity, or vascular injury, excessive NET formation or defective NET clearance can expose autoantigens, damage endothelium, and promote thrombosis (44–53). Consequently, a therapeutic conclusion cannot be drawn from the direction of NETosis alone. A key next step is to distinguish antimicrobial NET formation from pathological NET persistence, use primary human neutrophils and neutrophil-specific models, and test whether site-specific perturbation preserves migration and microbial killing while reducing tissue-damaging NET release. At present, neutrophil m^6^A biology should be described as an emerging field with several functional mechanisms but limited stage-resolved clinical validation.

### 3.3 Dendritic cells: a potentially amplifying compartment with limited subset-resolved evidence

Dendritic cells (DCs) translate innate sensing into adaptive immune instruction through antigen processing, costimulation, cytokine secretion, migration, and tolerance induction (60–63). A mechanistically informative study reported that METTL3-induced miR-338-3p in DCs promoted antigen-specific Th17 responses through regulation of DUSP16 (64). This axis links an m^6^A regulator in an antigen-presenting cell to a downstream T-cell phenotype and therefore provides more than a simple expression association.

Nevertheless, the DC literature remains narrow relative to the diversity of conventional DCs, plasmacytoid DCs, monocyte-derived DCs, and tolerogenic states. It is not yet clear whether the same m^6^A mechanism operates across subsets, whether it alters antigen processing versus costimulatory output, or how it affects the termination of type I interferon responses. Claims that m^6^A broadly controls DC-driven autoimmunity should therefore be qualified. The current evidence supports selected regulator–target pathways, but comprehensive conclusions about DC maturation, migration, cross-presentation, and tolerance require subset-specific loss-of-function models and disease-relevant human validation.

### 3.4 NK cells and ILCs: strong lineage evidence, limited inflammatory-disease validation

In NK cells, studies indicate that METTL3, YTHDF2, and mTOR-coupled m^6^A remodeling contribute to the regulation of activation, survival, antiviral function, and antitumor cytotoxicity (65–67). These studies provide strong evidence that m^6^A is functionally important for NK-cell effector programs. However, most were performed in infection or tumor models rather than chronic inflammatory diseases. It is therefore reasonable to conclude that m^6^A has an important role in NK-cell fitness, but premature to infer that increasing or decreasing a particular regulator will improve autoimmune or sterile inflammatory disease. The unresolved issue is whether inflammatory tissues use the same target RNAs and readers as tumor or viral contexts.

ILCs show similarly compelling lineage-specific biology. m^6^A regulation differs among ILC lineages, and the circZbtb20–ALKBH5–Nr4a1 pathway supports ILC3 homeostasis and function (68, 69). Because ILCs are enriched at intestinal, pulmonary, and cutaneous barriers, these mechanisms are relevant to local defense and repair (70–73). Yet direct links to chronic inflammatory outcomes remain limited. Future work should determine whether m^6^A regulates cytokine plasticity, tissue retention, and memory-like adaptation in ILC subsets during repeated injury. For both NK cells and ILCs, the evidence for basic cell biology is stronger than the evidence for inflammatory-disease intervention.

## 4 Adaptive immune cells: lineage stability, chronic activation, and immune memory

### 4.1 T cells: mechanistic evidence for cytokine sensing and lineage stability, with caution around exhaustion

T-cell responses require rapid expansion followed by contraction, memory formation, or restoration of tolerance. m^6^A can influence each step by controlling cytokine signaling, T-cell receptor (TCR) responses, lineage-defining transcripts, and survival. One of the better-characterized examples is the IL-7/STAT5/SOCS circuit: m^6^A-dependent turnover of SOCS-family transcripts permits appropriate IL-7/STAT5 signaling and T-cell homeostasis (108). This work provides mechanistic evidence that RNA decay removes negative-feedback transcripts and thereby permits cytokine responsiveness. It also illustrates why an m^6^A-mediated decrease in RNA abundance can be physiologically activating rather than suppressive.

Additional studies support subset-specific mechanisms. METTL14 sustains FOXP3 expression and induced Treg differentiation through an mTOR-related pathway (77), whereas WTAP-dependent methylation has been shown to support TCR signaling and T-cell survival (78). In activated CD4^+^ T cells, m^6^A has been reported to promote TNF mRNA degradation, providing a direct route for limiting cytokine output (79). FTO has been linked to Th1 differentiation and antimicrobial immunity, and to CD8^+^ T-cell survival through methylation-dependent regulation of Fas (80, 81). These findings argue against describing m^6^A as uniformly pro-inflammatory in T cells: the same regulatory layer can support effector competence, stabilize Treg identity, or accelerate inflammatory-transcript decay, depending on the target and subset.

Th17/Treg imbalance remains a clinically relevant framework in RA, SLE, IBD, and multiple sclerosis (74–76), but many reviews overextend indirect evidence by listing RORγt, FOXP3, STAT3, and cytokines without demonstrating direct m^6^A regulation of each transcript. A stronger interpretation is that m^6^A modifies the signaling and metabolic environment in which lineage decisions occur. The field now needs site-specific studies that test whether modifying a candidate RNA changes lineage stability after differentiation, not merely marker expression during acute activation.

CD8^+^ T-cell exhaustion requires particular caution. YTHDF2 and WTAP-dependent mechanisms can regulate CD8^+^ T-cell polyfunctionality or PD-1 translation in cancer models (82, 86, 87), and persistent antigen stimulation is also relevant to chronic infection (83–85). However, exhaustion-like states in autoimmune or sterile inflammatory disease may differ from tumor exhaustion in antigen load, tissue metabolism, and therapeutic objective. These oncology-derived mechanisms are valuable hypotheses, but they should not be presented as established drivers of inflammatory-disease persistence without direct validation in those settings.

### 4.2 B cells and plasma cells: rapidly expanding evidence with major subset and disease-context gaps

B cells contribute to autoimmunity through antibody production, antigen presentation, cytokine secretion, and the organization of memory and ectopic lymphoid responses (88–96). Recent studies have moved the m^6^A–B-cell field beyond general speculation. METTL14-dependent methylation appears important for an effective germinal-center B-cell response (97). In primary Sjögren’s syndrome, m^6^A modification of RRM2 has been linked to B-cell hyperactivity (98). In an SLE-related setting, YTHDF1 was reported to promote plasma-cell differentiation through IRF4 regulation (99). An ALKBH5/treRNA1/DDX46 pathway has also been implicated in BCR expression, although that mechanism was established in a neoplastic context and requires careful extrapolation to autoimmunity (100).

These studies indicate that m^6^A can act at distinct checkpoints—germinal-center selection, BCR expression, proliferative fitness, and plasma-cell differentiation. They do not yet establish a single unifying direction. Enhancing a germinal-center program could improve protective humoral immunity but worsen autoreactive affinity maturation; promoting plasma-cell differentiation may be beneficial during infection but harmful in antibody-mediated disease. Therefore, conclusions must specify the B-cell subset, antigenic context, and functional endpoint rather than refer to “B-cell activation” as one process.

The main limitations are the scarcity of subset-resolved human data, limited site-level validation, and insufficient linkage between m^6^A perturbation and antibody quality, autoantibody specificity, tissue deposition, or relapse. Resolution will require integrating single-cell BCR sequencing, plasma-cell and memory-cell profiling, site-resolved m^6^A mapping, and functional rescue. The B-cell field is clinically important and increasingly mechanistic, but it remains less mature than macrophage and T-cell research. The cell-type-specific roles of m6A regulation in innate and adaptive immune-cell inflammatory fate are summarized in **Table 1**.

**Table 1:** Cell-type-specific roles of m^6^A regulation in innate and adaptive immune-cell inflammatory fate.

Cell type	m^6^A regulator/axis	Direct target and RNA-fate mechanism	Functional outcome	Model or disease context	Evidence appraisal	Representative reference(s)
Macrophage	METTL3–STAT1	m^6^A in STAT1 CDS/3′UTR increases RNA stability/activity	Glycolysis and inflammatory polarization	IFN-γ/oxLDL; atherosclerotic inflammation	Mechanistically resolved	(28, 104, 107)
Macrophage	RBM4–YTHDF2–STAT1	Promotes YTHDF2-dependent decay of m^6^A-marked STAT1 RNA	Reduced glycolysis and M1-like activation	*In vitro* macrophage models	Mechanistically resolved	(105)
Macrophage	METTL3/YTHDF1–SLC37A2	Enhances SLC37A2 translation	Restrains LPS-driven glycolysis and intestinal inflammation	IBD mouse model	Mechanistically resolved	(26)
Macrophage	IGF2BP2–TSC1/PPARγ	Reader-dependent transcript stabilization	Controls macrophage activation and metabolic phenotype	Inflammatory disease models	Mechanistically resolved	(38)
Macrophage	METTL14–MyD88/NF-κB	Target/pathway regulation; site/reader chain incomplete	Inflammatory macrophage activation	Atherosclerosis and MASLD models	Functional/intermediate	(40, 41)
Macrophage	DDX5–TLR2/4	Modulates m^6^A abundance on TLR2/4 transcripts	Limits bacterial-infection-associated inflammation	Bacterial infection models	Functional/intermediate	(42)
Neutrophil	ALKBH5–G-CSFR/migration	Demethylase-dependent control of migration and G-CSFR expression	Antibacterial migration, granulopoiesis, mobilization	Infection models	Functional/intermediate	(54, 55)
Neutrophil	METTL3–SYK/ERK/MEK	m^6^A-dependent pathway linked to NET formation	Enhanced NETosis and lung injury	Acute lung injury	Functional/intermediate	(56)
Dendritic cell	METTL3–miR-338-3p–DUSP16	METTL3 induces miR-338-3p, regulating DUSP16	Promotes antigen-specific Th17 response	DC–T-cell model	Mechanistically resolved	(64)
NK cell	Reported METTL3/YTHDF2/mTOR–m^6^A axis	Proposed regulation of activation and effector-transcript RNA fate	Reported association with antiviral and antitumor fitness	Infection and cancer models	Functional/intermediate; disease relevance not yet established	(65–67)
ILC3	Reported circZbtb20–ALKBH5–Nr4a1 axis	Proposed ALKBH5-dependent effect on Nr4a1 mRNA fate	Reported contribution to ILC3 homeostasis and function	Barrier immune homeostasis model	Functional/intermediate; broader disease relevance not established	(68, 69)
T cell	m^6^A–SOCS–IL-7/STAT5	m^6^A-dependent decay of SOCS transcripts	Permits cytokine responsiveness and homeostasis	T-cell-specific genetic models	Mechanistically resolved	(108)
Treg	METTL14–FOXP3/mTOR	Supports FOXP3 expression and induced Treg differentiation	Treg stability/function	*In vitro* and immune models	Functional/intermediate	(77)
T cell	WTAP–TCR signaling	WTAP-dependent mRNA methylation controls TCR signaling/survival	T-cell activation and viability	T-cell genetic models	Mechanistically resolved	(78)
B cell	Reported METTL14–germinal-center axis	Proposed m^6^A-dependent effect on the germinal-center program	Reported contribution to affinity maturation/GC B-cell function	Immunization model	Functional/intermediate; direct target-level chain incomplete	(97)
B cell	Reported m^6^A–RRM2 association	Proposed m^6^A-linked effect on RRM2 RNA fate	Associated with B-cell hyperactivity	Primary Sjögren’s syndrome	Associative/emerging; direct target validation incomplete	(98)
Plasma cell	YTHDF1–IRF4	Reader-dependent regulation of IRF4	Promotes plasma-cell differentiation	SLE	Mechanistically resolved	(99)
B cell	Reported ALKBH5–treRNA1–DDX46 axis	Proposed regulation of BCR-related RNA processing/expression	Reported change in BCR availability	Neoplastic B-cell model	Functional/intermediate; autoimmune disease relevance not established	(100)

References listed are representative of the mechanisms summarized in each row and are not intended to be exhaustive.

Evidence appraisal criteria: Mechanistically resolved: direct target plus RNA-fate mechanism and supporting site/reader/rescue evidence; Functional/intermediate: regulator perturbation linked to a target pathway and phenotype, but with an incomplete causal chain; Associative/emerging: expression correlation or pathway inference without direct target validation. BCR, B-cell receptor; CDS, coding sequence; DC, dendritic cell; GC, germinal center; IBD, inflammatory bowel disease; IFN, interferon; ILC, innate lymphoid cell; MASLD, metabolic dysfunction-associated steatotic liver disease; NETosis, neutrophil extracellular trap formation; NK, natural killer; oxLDL, oxidized low-density lipoprotein; SLE, systemic lupus erythematosus.

## 5 Cross-cutting mechanistic axes: where m^6^A-sensitive pathways converge and diverge

**Figure 2:**
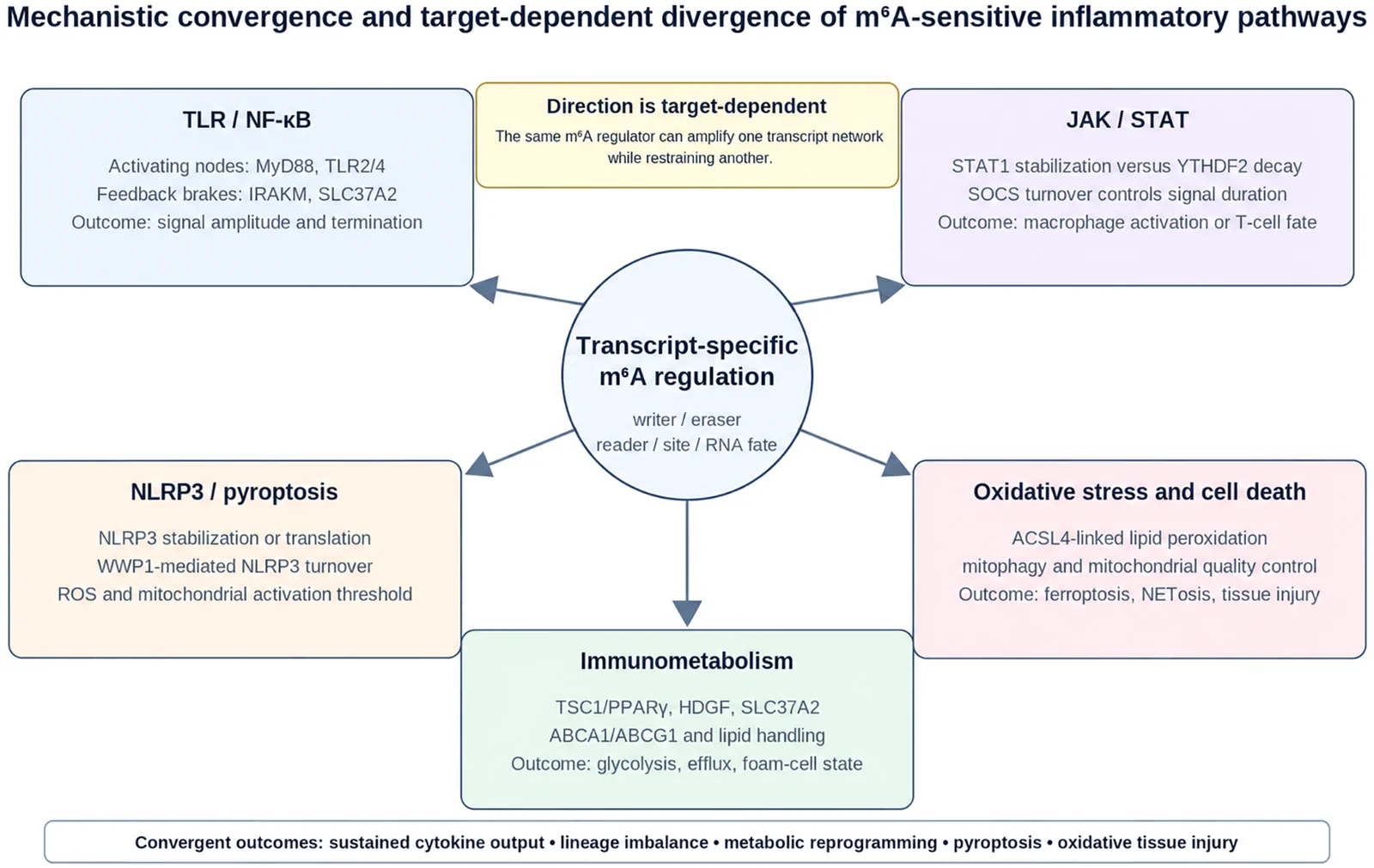
Mechanistic convergence and target-dependent divergence of m^6^A-sensitive inflammatory pathways. Transcript-specific m^6^A regulation can affect TLR/NF-κB signaling through activating components or feedback brakes; JAK/STAT signaling through STAT1 stability, YTHDF2-dependent decay, or SOCS turnover; NLRP3 inflammasome activity through direct RNA stabilization or translation, WWP1-mediated protein turnover, and mitochondrial or reactive-oxygen-species thresholds; immunometabolism through TSC1/PPARγ, HDGF, SLC37A2, ABCA1/ABCG1, and related lipid-handling programs; and oxidative cell death through ACSL4, mitophagy, and mitochondrial quality control. These axes converge on cytokine persistence, lineage imbalance, metabolic reprogramming, pyroptosis, NETosis, ferroptosis, and tissue injury. Direction is not intrinsic to an m^6^A regulator but depends on the regulated transcript network and cellular context.

The major m^6^A-sensitive inflammatory axes are summarized in **Figure 2**, illustrating how transcript-specific m^6^A regulation mechanistically converges on and diverges across TLR/NF-κB signaling, JAK/STAT signaling, NLRP3 inflammasome activation, immunometabolic programs, and oxidative stress–mediated cell death pathways.

The cell-centered evidence above shows that the same signaling pathway can be regulated at different RNA nodes in different immune cells. This section therefore does not repeat pathway descriptions; it compares how transcript-specific m^6^A events alter signal amplitude, feedback, metabolism, and inflammatory cell death. The key analytical point is that pathway activation alone is not evidence of a direct m^6^A mechanism. Directness requires identification of the modified RNA and the RNA-fate step that changes pathway behavior.

### 5.1 TLR/NF-κB signaling: activation versus termination

TLR/NF-κB signaling is shaped both by positive components such as TLR2/4, MyD88, TRAF6, and p65, and by termination mechanisms such as IRAKM, A20/TNFAIP3, SOCS proteins, and metabolic brakes (101). m^6^A can act on either side. METTL14-associated MyD88 regulation and METTL3-dependent NOD1 signaling support inflammatory amplification (40, 41, 102, 103), whereas DDX5-dependent modulation of TLR2/4 transcripts, METTL3/YTHDF1-dependent SLC37A2 translation, and regulation of IRAKM can restrain or terminate the response (26, 42, 43). Thus, global NF-κB activity is an endpoint; the mechanistic question is whether m^6^A increases the availability of an activating transcript or removes a negative-feedback constraint.

Several studies still infer an m^6^A–NF-κB mechanism from parallel changes in a regulator, global m^6^A abundance, and p65 phosphorylation. Such evidence is functional but incomplete. To resolve causality, future work should map the relevant site, identify the reader, quantify RNA half-life or translation, and show that a methylation-deficient target mutant abolishes the pathway phenotype. This standard is especially important because NF-κB can itself alter expression of m^6^A machinery, creating feedback that makes upstream and downstream relationships difficult to distinguish.

### 5.2 JAK/STAT signaling: transcript stability and negative-feedback control

The macrophage STAT1 and T-cell SOCS examples reveal two complementary m^6^A logics. In macrophage models, METTL3-mediated stabilization of STAT1 has been linked to sustained glycolysis and inflammatory activation, whereas YTHDF2-associated decay of STAT1 limits the same program (28, 104, 105). In T cells, m^6^A-dependent turnover of SOCS transcripts removes negative feedback and permits IL-7/STAT5 signaling (108). Therefore, the inflammatory direction cannot be predicted from whether m^6^A stabilizes or destabilizes RNA in the abstract. It depends on whether the target is a pathway activator or inhibitor and on whether sustained signaling is protective, pathogenic, or required for homeostasis in the relevant cell subset.

This framework also clarifies the interpretation of Th17/Treg studies. Persistent STAT3 signaling may favor pathogenic Th17 responses, whereas appropriate STAT5 and FOXP3 programs support Treg stability (74–78). However, pathway-level correlations should not be converted into direct target claims without transcript-specific evidence. The field now needs to distinguish immediate cytokine signaling from longer-term lineage maintenance and test the same m^6^A event at multiple stages of T-cell differentiation.

### 5.3 NLRP3 inflammasome and pyroptosis: direct RNA control and indirect stress thresholds

The NLRP3 inflammasome requires transcriptional priming and a second activation signal associated with ionic flux, mitochondrial injury, ROS, lysosomal damage, or metabolic stress (109–114). m^6^A can influence both layers. METTL14/IGF2BP2-dependent stabilization of NLRP3 and YTHDF1-mediated enhancement of NLRP3 translation provide direct routes to increased inflammasome competence (116, 117). By contrast, YTHDF1 can also increase WWP1, promoting NLRP3 ubiquitination and reducing caspase-1-dependent pyroptosis in sepsis (118). This apparent contradiction is a strong example of target-specific directionality within the same reader family.

Other reports link m^6^A regulators to mitochondrial injury, ROS, STING-related signaling, or metabolic remodeling that lowers the activation threshold of NLRP3 (115, 119–121). These mechanisms may be biologically important but should be distinguished from direct methylation of NLRP3 RNA. A rigorous study should separate effects on NLRP3 abundance from effects on the second activation signal and should assess whether changes in IL-1β release reflect inflammasome activation, altered transcriptional priming, or generalized cell injury.

### 5.4 HIF-1α, PPARγ, and immunometabolism: sustaining or resolving inflammatory states

Inflammatory macrophages often increase glycolysis through NF-κB/HIF-1α-linked programs, whereas PPARγ, fatty-acid oxidation, and cholesterol efflux are associated with lipid handling and, in some contexts, resolution (122–127). m^6^A connects these programs through transcript-specific effects rather than through a single metabolic switch. METTL3–STAT1 and METTL3/RBM15–Kdm6b mechanisms support glycolytic inflammatory activation (104, 106). IGF2BP2-mediated stabilization of TSC1 and PPARγ, FTO/ACSL4 regulation, and reported effects on ABCA1/ABCG1 or LXR-α pathways link m^6^A to lipid uptake, peroxidation, and cholesterol efflux (38, 128–131).

The principal limitation is that metabolic readouts are often interpreted too broadly. Increased extracellular acidification does not by itself prove an m^6^A-dependent glycolytic program, and altered lipid accumulation can reflect uptake, synthesis, oxidation, esterification, or efflux. Mechanistic studies should combine isotope tracing, transcript-specific methylation analysis, and functional rescue. They should also avoid assuming that “glycolysis” is always pathogenic or that PPARγ-linked programs are always reparative, because these functions vary across tissue-resident macrophages and disease stages.

### 5.5 Ferroptosis, mitophagy, oxidative stress, and NETosis: separate direct mechanisms from pathway intersection

Ferroptosis provides a comparatively direct connection between m^6^A and lipid-peroxidation-dependent cell death. FTO-mediated regulation of ACSL4 alters ferroptosis in LPS-stimulated macrophage inflammation (128). In contrast, many proposed links among m^6^A, mitophagy, ROS, pyroptosis, and NETosis are based on pathway co-occurrence rather than a mapped target RNA. ROS can connect mitochondrial dysfunction to inflammasome activation and NET formation, but this does not establish that every ROS-related phenotype is directly controlled by m^6^A (132–142).

Future work should distinguish three possibilities: direct modification of a cell-death transcript; indirect alteration of the metabolic or redox threshold that permits cell death; and secondary changes in m^6^A machinery caused by dying cells. Mitophagy is particularly context-dependent because removal of damaged mitochondria can restrain ROS and NLRP3 activation, whereas excessive or mistimed mitochondrial turnover may impair antimicrobial fitness. The most useful experiments will combine site-specific target perturbation with orthogonal measures of ferroptosis, pyroptosis, mitochondrial quality control, and NETosis rather than relying on a single marker.

## 6 Disease contexts: recurring immune programs and the maturity of supporting evidence

### 6.1 Autoimmune inflammation

RA, SLE, primary Sjögren’s syndrome, IBD, psoriasis, and multiple sclerosis involve different organs but repeatedly engage macrophage activation, NETosis, type I interferon responses, Th17/Treg imbalance, B-cell hyperactivity, and barrier or stromal dysfunction (143–158). m^6^A is relevant because it can regulate several of these programs at the RNA level. However, the maturity of evidence differs sharply. B-cell RRM2 and YTHDF1–IRF4 mechanisms provide disease-linked examples in primary Sjögren’s syndrome and SLE (98, 99), and the ALKBH5–LINC00968 axis has been linked to NETosis in RA (58). By contrast, many “m^6^A signatures” derived from bulk blood or synovial transcriptomes are associative and cannot identify the responsible cell type or establish causal methylation (31, 32, 143–147).

The most informative autoimmune model is therefore not a disease catalogue but a multicellular circuit. In RA, NETs, macrophages, and fibroblast-like synoviocytes can reinforce one another; in SLE, neutrophil-derived autoantigens, type I interferon, and B-cell/plasma-cell responses interact; in IBD, epithelial injury, macrophage activation, and T-cell imbalance form a barrier-centered loop (90, 93, 148–153). An m^6^A intervention should target a defined node within such a circuit rather than suppress an m^6^A regulator systemically. Patient studies must also distinguish whether an m^6^A change tracks disease activity, predicts treatment response, or persists independently as a mechanism of relapse.

### 6.2 Cardiometabolic inflammation

Atherosclerosis, obesity, diabetes, and MASLD/NASH expose immune cells to chronic lipid excess, insulin resistance, hypoxia, and oxidative stress (122, 123, 159–168). The best-characterized m^6^A evidence in these diseases is concentrated in macrophage state and metabolism: STAT1-dependent glycolysis, HDGF- or Kdm6b-linked activation, MyD88/NF-κB signaling, TSC1/PPARγ, cholesterol-efflux pathways, and ACSL4-associated ferroptosis (28, 38–41, 104–106, 128–131). These mechanisms support the concept that m^6^A can couple metabolic stress to persistent inflammatory behavior.

Nevertheless, cardiometabolic tissues contain hepatocytes, adipocytes, endothelial cells, smooth-muscle cells, fibroblasts, and multiple macrophage subsets. Bulk-tissue m^6^A changes cannot be assigned automatically to macrophages, and a macrophage mechanism demonstrated *in vitro* may not dominate disease progression *in vivo*. Stronger evidence will depend on using cell-type-specific perturbation and spatially resolved measurements to determine whether a candidate m^6^A axis acts during lesion initiation, foam-cell formation, inflammatory amplification, or resolution. Stage matters because promoting lipid handling may be beneficial in one phase but alter survival or repair in another.

### 6.3 Infection-associated hyperinflammation

In infection, inflammation must be strong enough to control pathogens but limited enough to prevent organ injury (169–181). m^6^A has been implicated in the regulation of antiviral RNA sensing, interferon responses, neutrophil migration, emergency granulopoiesis, and T-cell function (54, 55, 80, 176–184). The same mechanism can therefore be protective early and harmful later. ALKBH5-dependent neutrophil mobilization may improve antibacterial defense, whereas persistent neutrophil activation and NETosis can contribute to endothelial injury and immunothrombosis in sepsis (54, 55, 169–174).

This temporal reversal makes single time-point studies particularly difficult to interpret. Increased expression of an m^6^A regulator in late sepsis may represent pathogenic hyperinflammation, compensatory immune recovery, or a shift in circulating-cell composition. Longitudinal, cell-resolved sampling is needed to separate these possibilities. Therapeutic studies should measure pathogen burden and host-defense competence alongside cytokines and organ injury; otherwise, an apparently anti-inflammatory intervention may simply suppress immunity.

### 6.4 Barrier-tissue inflammation

The intestine, lung, and skin are continuously exposed to microbes, allergens, pollutants, dietary metabolites, and mechanical stress (70, 185–199). In these organs, immune-cell m^6^A regulation cannot be separated from epithelial resilience, stromal responses, microbiota-derived signals, and tissue repair. Disease-linked examples include KLF4 and TRADD regulation in intestinal barrier dysfunction, YTHDF1–CLOCK signaling in allergic airway inflammation, and FTO-targeted epithelial interventions (191–195). These findings indicate that an m^6^A-dependent inflammatory phenotype may arise in non-immune cells and secondarily reshape immune recruitment.

Barrier disease therefore provides an important test of the “immune-cell inflammatory fate” concept. A macrophage or ILC program may be maintained because the epithelium continues to release danger signals, while epithelial injury may persist because immune-cell cytokines and NETs prevent repair. Future models should preserve these reciprocal interactions—for example, organoid–immune co-cultures, tissue slices, and spatial multi-omics—rather than study isolated cell types alone. Claims about barrier inflammation should specify whether the primary m^6^A event occurs in an immune, epithelial, or stromal compartment. The major m⁶A-sensitive inflammatory programs linking RNA-level regulation to disease contexts and translational implications are summarized in **Table 2**.

**Table 2:** Major m^6^A-sensitive inflammatory programs linking RNA-level regulation to disease contexts and translational intervention.

Program/context	Most supported m^6^A-sensitive nodes	Predominant cell compartments	Context-dependent interpretation	Major evidence gap/translational implication
TLR/NF-κB	MyD88^F^, TLR2/4^F^, NOD1^F^, IRAKM^F^, SLC37A2^D^ (26, 40–43, 102, 103)	Macrophages; epithelial/innate cells	m^6^A can amplify activating transcripts or support feedback termination.	Resolve target sites/readers and separate upstream m^6^A effects from NF-κB-driven feedback.
JAK/STAT	STAT1 stability/decay^D^; SOCS turnover^D^; FOXP3-linked programs^F^ (77, 104, 105, 108)	Macrophages and T cells	Direction depends on whether the target is a pathway activator or inhibitor and on lineage stage.	Test matched axes across acute signaling, differentiation, and long-term state maintenance.
NLRP3/pyroptosis	NLRP3 stabilization/translation^D^; WWP1-mediated NLRP3 turnover^D^ (116–119)	Macrophages/microglia; injured tissues	The same reader can enhance or restrain pyroptosis through different target RNAs.	Distinguish direct NLRP3 RNA regulation from indirect ROS/mitochondrial effects.
Immunometabolism	STAT1^D^, Kdm6b^F^, TSC1/PPARγ^D^, HDGF^F^, ABCA1/ABCG1^F^, ACSL4^D^ (38, 39, 104, 106, 128–131)	Macrophages; metabolic tissues	Glycolysis or lipid handling can be adaptive or pathogenic depending on subset and stage.	Use isotope tracing, cell-specific models, and target rescue rather than surrogate metabolic markers alone.
Autoimmune inflammation	NETosis axes^F^; B-cell RRM2^F^; YTHDF1–IRF4^D^; transcript-resolved macrophage/T-cell programs^D^; broader disease signatures^A^ (58, 77, 98, 99)	Neutrophils, macrophages, T/B cells, stroma	Different immune circuits dominate RA, SLE, pSS, IBD, psoriasis, and MS.	Prioritize cell-resolved mechanisms and prospectively validated prediction over bulk regulator signatures.
Infection/sepsis	ALKBH5-dependent neutrophil responses^F^; antiviral transcript-level mechanisms^D^; transcript-resolved NLRP3 axes^D^; broader sepsis signatures^A^ (54, 55, 116–118, 176–184)	Neutrophils, macrophages, DCs, T/NK cells	Protective early host defense can become injurious hyperinflammation later.	Longitudinal studies must measure pathogen control, immune suppression, and organ injury together.
Barrier-tissue inflammation	KLF4^F^, TRADD^F^, YTHDF1–CLOCK^D^, epithelial FTO pathways^F^ (191–195)	Epithelial cells plus macrophages, ILCs, neutrophils, T cells	The primary m^6^A event may be epithelial rather than immune-cell intrinsic.	Use organoid–immune co-culture and spatial analysis to resolve reciprocal cell interactions.
Therapeutic translation	Regulator inhibitors^F^; target-RNA/site strategies^A^; reader modulation^A^; targeted delivery^F^ (200–213)	Cell- and tissue-specific	Global modulation risks infection, hematopoietic effects, impaired repair, and tumor-related consequences.	Demonstrate cell-specific target engagement, stage-appropriate benefit, and preservation of protective immunity.

Evidence notation: ^D^direct transcript-level evidence identifying a target RNA and corresponding RNA-fate mechanism; ^F^functional/intermediate evidence linking regulator perturbation to a pathway or phenotype but leaving part of the target–reader–RNA-fate chain unresolved; ^A^associative/emerging evidence based mainly on expression, global m^6^A, pathway-level correlations, or translational proposals. Assignments reflect mechanistic resolution within the evidence synthesized in this review and are not formal study-quality scores.

DC, dendritic cell; IBD, inflammatory bowel disease; ILC, innate lymphoid cell; MASLD, metabolic dysfunction-associated steatotic liver disease; MS, multiple sclerosis; pSS, primary Sjögren’s syndrome; RA, rheumatoid arthritis; ROS, reactive oxygen species; SLE, systemic lupus erythematosus.

## 7 Translational implications: from global m^6^A modulation to target-RNA and cell-state precision

m^6^A regulators are attractive drug targets because they sit upstream of multiple inflammatory outputs, but this same breadth creates safety risk. METTL3, METTL14, FTO, ALKBH5, and YTH-family proteins have important roles in hematopoiesis, immune development, metabolism, tissue repair, and tumor biology (7, 20, 200–206). Systemic inhibition may therefore suppress a pathogenic transcript in one cell while impairing protective immunity or regeneration in another. The therapeutic objective should not be to “increase” or “decrease” global m^6^A. It should be to alter a defined regulator–target–reader mechanism in a defined cell state and disease phase.

Target-RNA precision can be approached at several levels: blocking a regulator–substrate interaction, editing or masking a disease-relevant m^6^A site, modulating the responsible reader, or delivering an RNA construct that restores the desired target output. Each strategy requires proof that the selected transcript is both causal and sufficiently restricted to avoid broad perturbation. A regulator that modifies thousands of RNAs is a weak therapeutic target unless the delivery system or molecular design narrows its action.

Cell-specific delivery is therefore part of the mechanism, not merely a formulation issue. Lipid nanoparticles, antibody-targeted nanoparticles, engineered exosomes, biomimetic coatings, and neutrophil- or macrophage-directed systems are being developed for immune modulation (207–213). Their limitations include incomplete tissue enrichment, uptake by unintended cells, altered biodistribution during inflammation, immunogenicity after repeated dosing, and uncertain durability. For m^6^A-targeted therapy, even modest off-target delivery may have complex effects because the same regulator can control different transcript networks in macrophages, lymphocytes, epithelial cells, and fibroblasts.

Timing is equally important. During early infection, m^6^A-dependent interferon responses, neutrophil migration, and effector T-cell survival may be protective; during later hyperinflammation, persistent cytokine output, NETosis, or pyroptosis may become injurious (12, 54, 169–181, 214, 215). In chronic autoimmunity, the relevant target may be a stable Th17, plasma-cell, macrophage, or NETosis-active state, whereas during resolution the priority is to preserve Treg function, efferocytosis, barrier repair, and antimicrobial competence. Dose and treatment duration should therefore be selected against a stage-specific biological objective, not only against a molecular target.

m^6^A signatures may also aid patient stratification, but current signatures are often derived from bulk expression of m^6^A regulators rather than direct methylation or target-RNA activity (31–33, 216, 221). A clinically useful biomarker should identify a reproducible cell state, link to a functional transcript network, predict an outcome beyond standard clinical variables, and be validated prospectively. Integration with single-cell transcriptomics, spatial profiling, proteomics, metabolomics, and clinical phenotypes may distinguish upstream drivers from downstream markers (217–220). Without that distinction, an m^6^A signature may classify inflammation but remain therapeutically unactionable.

## 8 Discussion and conclusion

The central conclusion of this review is that m^6^A is best understood as a context-dependent regulator of RNA fate rather than a universal pro-inflammatory or anti-inflammatory switch. The most mechanistically developed evidence comes from transcript-resolved mechanisms in macrophages and T cells, including STAT1 stability and decay, SOCS turnover, SLC37A2 translation, TSC1/PPARγ stabilization, and TCR- or FOXP3-linked programs. Neutrophil, DC, NK-cell, ILC, and B-cell studies now include several direct mechanisms, but their breadth, disease validation, and stage resolution remain uneven. Presenting these fields as equally mature would overstate the literature.

Methodological limitations explain much of the uncertainty. Expression of a writer, eraser, or reader does not measure methylation activity. Global m^6^A assays cannot identify the responsible transcript. MeRIP-seq has limited resolution and is influenced by antibody performance and RNA abundance. Differences in resolution, antibody dependence, sequence constraints, and validation scope among MeRIP-seq, miCLIP, SELECT, SCARLET, MazF-based approaches, and direct RNA sequencing may partly explain inconsistent target-site assignments across studies. Bulk tissues confound cell composition with cell-intrinsic regulation. Overexpression or knockdown may alter many transcripts simultaneously, and changes in cytokines or pathway phosphorylation do not prove a direct m^6^A target. Conditional genetic models can also produce developmental compensation that differs from acute pharmacological inhibition. These limitations should be acknowledged when assigning causal language.

A minimum causal framework for future studies should include: (i) identification of the target RNA and modified site or region; (ii) orthogonal validation of methylation; (iii) measurement of the relevant RNA-fate process, such as half-life, translation, splicing, or localization; (iv) demonstration of reader dependence when applicable; (v) rescue with the target RNA or a methylation-deficient mutant; (vi) validation in primary cells and cell-specific *in vivo* models; and (vii) linkage to a disease-relevant functional outcome. For heterogeneous tissues, single-cell or spatial approaches should be integrated with targeted m^6^A measurements rather than used as substitutes for them.

Conflicting results should be treated as data to be explained, not selectively excluded. Differences may arise from the target transcript, site location, reader abundance, cell subset, activation state, disease stage, sex, metabolic environment, or experimental model. A useful future direction is to test the same regulator across matched cell types and time points while measuring its target repertoire. Such studies could reveal whether an m^6^A axis initiates inflammation, maintains a pathological state, or supports recovery after injury.

Translation will require an even higher standard. Candidate therapies should demonstrate target engagement in the intended cell, preservation of host defense and tissue repair, and benefit across disease-relevant stages. Biomarker programs should move from regulator-expression signatures to functional target-RNA networks. With these constraints, m^6^A research can contribute more than another list of inflammatory molecules: it can provide a mechanistic framework for understanding why selected immune-cell states persist and how they might be reprogrammed without globally suppressing immunity.
